# Phenotypic and genotypic characterization of antimicrobial susceptibility of avian pathogenic *Escherichia coli* isolated from broiler chickens

**DOI:** 10.14202/vetworld.2017.1167-1172

**Published:** 2017-10-03

**Authors:** Gamal Younis, Amal Awad, Nada Mohamed

**Affiliations:** Department of Bacteriology, Mycology and Immunology, Faculty of Veterinary Medicine, Mansoura University, El Mansoura, 35516, Egypt

**Keywords:** antimicrobial resistance, broilers, *Escherichia coli*, multiplex polymerase chain reaction, resistant genes

## Abstract

**Aim:**

Avian pathogenic *Escherichia coli* (APEC) is pathogenic strains of *E. coli* that are responsible for one of the most common bacterial diseases affecting poultry worldwide. This study was designed to determine the occurrence, antibiotic resistance profile, and antibiotic resistance genes of *E. coli* isolated from diseased and freshly dead broilers.

**Materials and Methods:**

In that context, a total of 200 broilers samples were examined by standard microbiological techniques for isolation of *E. coli*, and tested for their antimicrobial susceptibility against 15 antimicrobial agents using disc diffusion method. In addition, *E. coli* isolates were screened by multiplex polymerase chain reaction for detection of a number of resistance genes including *aadA1* gene encodes streptomycin/neomycin, *tetA* encodes resistance to tetracycline, sul1 encodes sulfonamides, and β-lactamase encoding genes (bla_TEM_ and bla_SHV_).

**Results:**

A total of 73 (36.5%) isolates were biochemically identified as *E. coli* strains. O78, O2, and O1 are the most prevalent serotypes detected. *E. coli* displayed a high resistance against penicillin (100%), followed by cefepime (95.8%) and a low resistance to norfloxacin (36.9%), and chloramphenicol (30%). Depending on the results of PCR, *sul*1 gene was the most predominant antibiotic resistant gene (87%) followed by bla_TEM_ (78%), *tetA* genes (60%), and *aadA* (54%). However, bla_SHV_ had the lowest prevalence (23%).

**Conclusion:**

The obtained results demonstrated the importance of studies on APEC and antibiotic resistance genes in our region which associated with intensive poultry industry, aiming to acquire preventive measures to minimize losses due to APEC and associated multidrug-resistance and resistance genes that of high significance to the rational use of antibiotics in clinical and public health.

## Introduction

Avian pathogenic *Escherichia coli* (APEC) is a subgroup of extraintestinal pathogenic *E. coli*, enters through different routes including genital and respiratory tracts and causes various extraintestinal diseases collectively known as collibacillosis in chickens, which are responsible for high economic losses in the chicken industry [1]. The pathogenicity of APEC allows certain intestinal commensal *E. coli* to become APEC [2]. APEC belongs mostly to the serotypes O1, O2, and O78 or is often nontypable. APEC is also suspected to be a potential zoonotic risk for human [3].

Treatment of diseases caused by *E. coli* usually necessitates antimicrobial chemotherapy. The decision of using antimicrobial therapy based on the microorganism susceptibility and the drug pharmacokinetics for obtaining the required therapeutic concentration at the site of infection and therefore clinical efficacy [4]. Therefore, veterinarians have a restricted choice of antimicrobial agents to use it in the poultry industry, due to problems of multidrug-resistance (MDR) and human health hazard. Moreover, the constant misuse of antimicrobials led to increase rate of antibiotic resistance [5]. Antibiotic resistance is a major problem that threatens human and animal health especially in underdeveloped and developing countries where antibiotics are used without control for prophylaxis and treatment of human and animal illnesses [6]. *E. coli* present in both human and animals possess resistance to several classes of antibiotics such as aminoglycosides, penicillin, streptomycin, cephalosporins, sulfonamides, tetracycline, and quinolones [7].

Many drug-resistant strains and genes can be transmitted and disseminated between animal and human pathogens, which not only increases the difficulty in treating animal diseases but also threatens the human health [8]. Therefore, the main goal of this study was to, determine the antibiotic resistance profile of APEC isolates and to detect their associated antibiotic resistance genes.

## Materials and Methods

### Ethical approval

There is no ethical approval necessary.

### Samples collection

In this study, a total of 200 chicken visceral organs (liver, lungs, heart, spleen) and intestinal contents were collected randomly from diseased and freshly dead chicken broilers from different poultry farms located in the district of Mansoura City, Egypt. The chickens were ranged from 35 to 50 days old and showed different forms of diarrhea. The predominant lesions revealed in postmortem examination were ascites, pericarditis, splenitis, perihepatitis, airsacculitis, and peritonitis. Using sterile scissors and tissue forceps, the visceral organs from each bird were collected separately and put in a polyethylene bag and transferred immediately in an ice tank to the laboratory for bacteriological analysis.

### Bacteriological examination

From each chicken visceral organ, 2 g was directly inoculated in MacConkey broth and incubated for 18 h at 37°C. Then, a loopful from the previously inoculated broth was streaked onto MacConkey agar (Oxoid) plates for 24 h at 37°C. Rose pink colonies were picked up and streaked onto Eosin Methylene Blue (Oxoid) and incubated overnight at 37°C. The identification of *E. coli* isolates depends on the colonies morphological characters, and biochemical tests results following Ewing [9]. Further identification of *E. coli* isolates was done using commercial biochemical test kits (bioMerieux API, France).

### Serotyping

Serological identification of *E. coli* isolates was done using rapid diagnostic *E. coli* antisera sets (Denka Seiken Co., Japan) according to Kok *et al*. [10] at the Department of Food Hygiene Control, University of Benha, Egypt.

### Antimicrobial sensitivity testing

It was performed by disc diffusion method using Muller-Hinton agar using 15 antibiotic disc belongs to seven different antimicrobial classes including sulfamethoxazole (100 µg/disk), levofloxacin (5 µg/disk), chloramphenicol (30 µg/disk), norfloxacine (10 µg/disk), tetracycline (30 µg/disk), streptomycin (10 µg/disk), cefoxitin (30 µg/disk), ampicillin/sulbactam (10 µg/disk), neomycin (30 µg/disk), doxycycline (30 µg/disk), cefepime (30 µg/disk), cefotaxime (30 µg/disk), penicillin (10 µg/disk), amoxicillin (10 µg/disk), and nalidixic acid (30 µg/disk). Interpretation of the results was done following Clinical and Laboratory Standards Institute Guidelines [11].

### DNA extraction

DNA was extracted according to Ramadan *et al*. [12] briefly; three presumptive *E. coli* colonies were inoculated into 3 ml trypticase soy broth and incubated for 18 h at 37°C. 1 ml of the previously inoculated broth was centrifuged at 8,000 g for 2 min. The sediment was washed with DNase/RNase-free water and heated at 95°C for 15 min; the supernatants were used as DNA template.

### Detecting antimicrobial resistance genes by multiplex PCR

Multiplex PCR assay was done targeting five antibiotic resistance genes (bla_TEM_, bla_SHV_, TetA(A), *Aada2*, and *sul1*) of APEC isolates. Primers used for multiplex PCR are as per the previously reported researchers [13-16]. PCR was performed in a total volume of 50 µL consisting of 25 µL of 2X PCR Master Mix (Promega, Madison, USA), 1 µL of each primer (Metabion, Germany), and 6 µL DNA templates. After an initial denaturation at 94°C for 5 min, 35 amplification cycles consisting of denaturation at 94°C for 30 s, annealing at 54°C for 45 s, and extension at 72°C for 45 s per kbp were performed, followed by a final extension at 72°C for 10 min. Amplified genes were separated by electrophoresis in a 1.5% agarose gel. The separated PCR products were visualized under ultraviolet light and photographed.

## Results

Out of 200 examined specimens, *E. coli* was identified in 36.5% (73/200) of the total examined samples based on morphological and biochemical characteristics. The recovery rate of *E. coli* from different chicken samples was 28.76%, 27.39%, 23.28%, 15.06%, and 5.46% form lungs, spleen, heart, liver, and intestinal contents, respectively (Table-1). *E. coli* isolates were serotyped into 26 serotypes including O1, O2, O78, O26, O153, O114, O91, O121, O44, O63, O158, O171, O146, O124, O15, O8, O145, O117, O166, O128, O111, O55, O119, O159, O6, and O126 (Table-2). O78, O1, and O2 were the most prevalent serotypes with an incidence of 17.8%, 9.5%, and 9.5%, respectively.

**Table-1:** Number of strains of avian pathogenic *E. coli* isolated from different samples.

Organs	Number of isolated strains n=73 (%)
Lungs	21 (28.76)
Liver	11 (15.06)
Spleen	20 (27.39)
Intestinal contents	4 (5.47)
Heart	17 (23.28)

E. coli=Escherichia coli

**Table-2:** Serogroups of avian pathogenic *E. coli.*

Number of strains (n=73)	Type of samples	Serodiagnosis	Percentages
13	Lungs (4), spleen (4), heart (3), intestinal content, liver	O78	17.8
7	Lungs (2), spleen (2), heart, liver, intestinal content	O2:H6	9.5
7	Lungs (2), heart (3), spleen (2)	O1:H7	9.5
4	Heart (2), lungs, liver	O91:H21	5.4
4	Spleen, heart, lungs (2)	O8:H21	5.4
3	Spleen, lungs, liver	O114:H4	4
3	Lungs (2), heart	O126:H21	4
3	Heart, spleen, lungs	O26:H11	4
3	Spleen, liver, lungs	O145	4
3	Spleen, liver, heart	O44:H18	4
2	Intestinal contents, liver	O166	2.7
2	Lungs, heart	O117:H7	2.7
2	Intestinal contents, lungs	O55:H7	2.7
2	Liver (2)	O111:H2	2.7
2	Heart, spleen	O124	2.7
2	Spleen, heart	O158	2.7
2	Lungs, heart	O128:H2	2.7
1	Lungs	O153:H2	1.3
1	Liver	O119:H6	1.3
1	Spleen	O63	1.3
1	Spleen	O15:H2	1.3
1	Lungs	O159:H21	1.3
1	Spleen	O171:H2	1.3
1	Spleen	O146:H21	1.3
1	Lungs	O6:H4	1.3
1	Liver	O121:H7	1.3

E. coli=Escherichia coli

Phenotypically, the recovered *E. coli* strains were tested for their antimicrobial resistance against 15 antimicrobial agents (Table-3). Resistance was most frequently detected against penicillin (100%) followed by amoxicillin (94.5%), cefepime (95.8%), cefoxitin (90.4%), cefotaxime (76.7%), neomycin (89%), sulfamethoxazole (90.4%), streptomycin (73.9%), doxycycline (69.8%), tetracycline (53.4%), nalidixic acid (49.3%), ampicillin/sulbactam (46.5%), levofloxacin (42.4%), and to lesser extents chloramphenicol (30%), and norfloxacin (36.9%). All *E. coli* isolates displayed a MDR to antimicrobial agents (Table-4).

**Table-3:** Antibiograms of isolated *E. coli* strains.

Antimicrobial class	Antimicrobial agent	*E. coli* n=73 (%)
β-lactams	Amoxicillin	69 (94.5)
	Ampicillin/sulbactam	34 (46.5)
	Penicillin	73 (100)
Cephalosporins	Cefepime	70 (95.8)
	Cefoxitin	66 (90.4)
	Cefotaxime	56 (76.7)
Sulfonamides	Sulfamethoxazole	66 (90.4)
Aminoglycosides	Neomycin	65 (89)
	Streptomycin	54 (73.9)
Tetracycline	Tetracycline	39 (53.4)
	Doxycycline	51 (69.8)
Phenicols	Chloramphenicol	22 (30)
Quinolones	Levofloxacin	31 (42.4)
	Norfloxacin	27 (36.9)
	Nalidixic acid	36 (49.3)

E. coli=Escherichia coli

**Table-4:** Antimicrobial resistance patterns and resistance genes profiles of *E. coli* strains.

Number of strains	Strain antibiotic phenotypes	Resistance genes identified
3	FEP, N, SMZ, NOR, NA, AX, P, CTX	*bla*_TEM_, *aada*2, *sul*1
4	FEB, TE, N, S, DO, NA, AX, P, CTX	*bla*_SHV_, *tetA* (A), *aad*A2
1	FEP, FOX, S, NA, AX, P, CTX	*bla*_SHV_
2	FEB, FOX, DO, NA, AX, S, SAM, P, CTX	*bla*_TEM_, *tetA* (A)
5	FEP, TE, FOX, S, SMZ, DO, NA, AX, SAM, P, CTX	bla_TEM_, *tetA* (A), sul1
2	FEB, TE, N, FOX, S, SMZ, DO, AX, P, CTX	*tet*A (A), *sul*1
6	FEB, N, FOX, SMZ, DO, AX, P	*bla*_TEM_, *tetA* (A), *sul*1
5	FEB, N, FOX, SMZ, C, S, LEV, NOR, AX, SAM, P, CTX	*bla*_TEM_, *aada*2, *sul*1
2	FEB, TE, N, FOX, SMZ, DO, C, NA, AX, P	*sul*1
3	FEP, N, FOX, S, SMZ, LEV, NOR, AX, SAM, P, CTX	*bla*_SHV_, *bla*_TEM_, *tet*A (A), *aad*A2, *sul*1
2	FEB, TE, N, FOX, S, SMZ, DO, C, LEV, NOR, AX, P, CTX	*Tet*A (A), *sul*1
5	FEB, N, FOX, S, SMZ, NOR, AX, SAM, P, CTX	*bla*_TEM_, *aada*2, *sul*1
2	FEB, TE, N, FOX, S, SMZ, DO, LEV, NA, SAM, AX, P, CTX	*bla*_SHV_, *tetA* (A)
4	FEB, N, FOX, S, SMZ, DO, LEV, NA, AX, P, CTX	*bla*_TEM_, *aada*2, *sul*1
4	FEP, TE, N, FOX, SMZ, DO, AX, SAM, P, CTX	*bla*_SHV_, *bla*TEM, *aada*2, *sul*1
5	FEB, TE, N, FOX, SMZ, DO, AX, P, CTX	*bla*_TEM_, *tetA* (A) *aada*2, *sul*1
3	TE, N, FOX, S, SMZ, DO, C, NOR, NA, AX, SAM, P, CTX	*bla*_SHV,_ *tetA* (A), *aada*2, *sul*1
5	FEB, N, FOX, S, SMZ, LEV, AX, P	*bla*_TEM_, *sul*1
6	FEB, TE, N, FOX, S, SMZ, DO, C, LEV, NOR, NA, AX, SAM, P, CTX	*bla*_TEM_, *tetA* (A), *sul*1
4	FEB, TE, N, FOX, S, SMZ, DO, C, LEV, NA, SAM, P	*bla*_TEM_, *tetA* (A), *aada*2, *sul*1

E. coli=Escherichia coli

**Figure-1:**
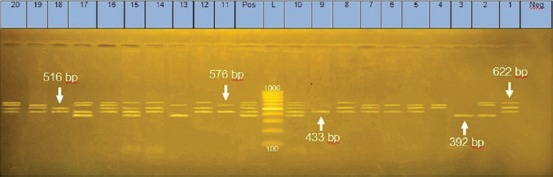
Agarose gel electrophoresis of multiplex PCR showing amplification of five antimicrobial resistance genes, *bla*_TEM_ at 516 bp, *sul*1 at 433 bp *aad*AA1 at 622 bp, *tetA* at 576 bp and *bla*_SHV_ at 392 bp, Lane L: DNA molecular size marker (1000 bp), Lane Neg: Negative control, Lane Pos: Positive control.

Multiplex PCR was used for detection of antibiotic resistance genes including bla_TEM,_ bla_SHV,_ *aadA, tetA*, and *sul1* (Figure-1). The recovery rate of these antibiotic resistance genes was 78%, 23%, 54%, 60%, and 87%, respectively. The *sul1* gene was the most prevalence gene (87%). In contrast, bla_SHV_ had the lowest prevalence (23%) as shown in Table-4.

## Discussion

*E. coli* is normally inhabitant the intestinal tract of poultry, but specific strains known as APEC have specific virulence factors and are able to cause avian colibacillosis. This disease is a major problem in poultry industry, as it causes high economic losses. Among 200 tested chicken samples, 73 (36.5%) *E. coli* isolates were recovered. These findings were nearly similar to the findings of Momtaz and Jamshidi [17]. On the other hand, Kiliç *et al*. [18] recorded a higher recovery rate (48%) of *E. coli* out a total of 100 examined chicken samples. In Egypt, 49 (20%) *E. coli* strains out of 242 samples were isolated [19], which is relatively lower than our findings.

Serologically, APEC isolates usually belong to certain O serogroups, especially O1, O2, O8, O15, O18, O35, O78, O88, O109, and O115 [20]. As first demonstrated by Sojka and Carnaghan [21], O1, O2, and O78 are the most frequently isolated from colibacillosis in the many countries worldwide, that proven their role as particularly adapted pathogens that allow involvement in extraintestinal infections [22]. In this study, twenty six O serogroups were identified among the 73 APEC isolates. Among the isolates that could be typed, the most prevalent serogroups were O78, O2 and O1 with a prevalence of 17.8%, 9.5%, and 9.5%, respectively. In many studies conducted in Egypt, nearly the same serotypes with a predominance of O78 have been identified [12,23,24].

Antimicrobial agents are used in the prevention and treatment of infections and can also use as growth promoting agents in animals. Under the pressure of antibiotic selection, MDR bacteria have been aroused. In this study, *E. coli* showed a high rate of resistance to most antimicrobials tested. 100% of the tested *E. coli* isolates showed resistance against penicillin, 95.8% to cefepime and 94.5% to amoxicillin followed by considerable resistance to the rest of the examined agents. Most of these antimicrobials are regularly used as growth promoters or as prophylactic agents in the poultry industry in Egypt [25,26] and concurred with the previous reports [27-32].

Many studies have been reported the presence of antibiotic resistance genes in APEC strains [33]. The resistance genes mediated by plasmid can make the resistance prevail among various bacteria that lead to acquiring resistance genes without difficulty and produce MDR [34,35].

Phenotypic multi-resistance of *E. coli* isolates to aminoglycoside, β-lactams, tetracycline, and sulfonamides antibiotics could be attributed to the presence of *aadA*, bla_TEM_*, tetA(A)*, and *sul1* resistance genes, respectively, among the tested isolates. Regarding the distribution of these antibiotic resistance genes among *E. coli* isolates, *sul*1 was detected in 64 (87%) isolates and bla_TEM_ identified in 57 (78%) isolates meanwhile *tetA* gene was identified in 44 (60%) isolates, 40 (54%) isolates were harbored *aadA*, and 17 (23.28%) isolates were carried bla_SHV_. These findings agree with Ammar *et al*. [19] who found that bla_TEM_ and *sul*1 genes had the highest prevalence among the tested antibiotic resist genes which have being amplified in all tested isolates (100%). In addition, bla_TEM_ was detected in most of β-lactams resistance strains, similar results were reported by Ali [36]. In contrast to our results, a relatively higher prevalence of bla_SHV_ was previously recorded in Spain (88%) [13] and Egypt (79%) [37]. These results are signifying that the results of antibiotic disc diffusion test actually agreed with the results of PCR for detection of the relevant antibiotic resistance genes.

The risk of spreading antibiotic resistance genes to humans should be considered when there is contamination of animal products, especially chickens, by bacterial strains resistant to most of antibiotics [38]. Recently, many of extended-spectrum beta-lactamases (ESBL) producing *E. coli* have become a worldwide issue. The ESBL-positive *E. coli* strains are highly resistant to a wide range of antibiotics. Controlling such strains with usually used antibiotics is ineffective; recently, there are few antibacterial alternatives that remain effective against these MDR pathogens [39].

## Conclusion

It is very important to control APEC because it represents a grave danger to poultry and is a potential source of transferring MDR genes to human-specific *E. coli* or other bacteria such as *Staphylococcus aureus* and *Shigella* strains. The fact that this pathogen is naturally present in daily consumed food should be considered as a serious public health and food biosafety.

## Authors’ Contributions

GY design the study shared in data analysis and revised the manuscript. AA and NM shared in samples collection, performing the tests, manuscript writing and data analysis. All authors read and approved the final manuscript.
